# A133 MARGIN THERMAL ABLATION WITH SNARE-TIP SOFT COAGULATION EFFECTIVELY MITIGATES RECURRENCE AFTER ENDOSCOPIC MUCOSAL RESECTION OF LARGE NON-PEDUNCULATED COLORECTAL POLYPS

**DOI:** 10.1093/jcag/gwad061.133

**Published:** 2024-02-14

**Authors:** S X Jiang, A Zarrin, A Walia, C Galorport, W Xiong, R Enns, E Lam, N Shahidi

**Affiliations:** The University of British Columbia Faculty of Medicine, Vancouver, BC, Canada; The University of British Columbia Faculty of Medicine, Vancouver, BC, Canada; St. Paul’s Hospital, Division of Gastroenterology, University of British Columbia, Vancouver, BC, Canada; St. Paul’s Hospital, Division of Gastroenterology, University of British Columbia, Vancouver, BC, Canada; University of British Columbia, Department of Pathology and Laboratory Medicine, Vancouver, BC, Canada; St. Paul’s Hospital, Division of Gastroenterology, University of British Columbia, Vancouver, BC, Canada; St. Paul’s Hospital, Division of Gastroenterology, University of British Columbia, Vancouver, BC, Canada; St. Paul’s Hospital, Division of Gastroenterology, University of British Columbia, Vancouver, BC, Canada

## Abstract

**Background:**

Recurrence following endoscopic mucosal resection (EMR) historically occurs in approximately 15-20% of large (≥20 mm) non-pedunculated colorectal polyps (LNPCPs). Margin thermal ablation with snare-tip soft coagulation (STSC) of the post-EMR defect is an evidence-based modality to mitigate recurrence. However, international validation of margin thermal ablation outcomes is needed.

**Aims:**

To evaluate the frequencies of endoscopic and histologic recurrence following margin thermal ablation with STSC for LNPCPs managed by EMR.

**Methods:**

Consecutive patients ampersand:003E 18 years of age who underwent endoscopic resection for a LNPCP were enrolled in a prospective single center observation cohort study (clinicaltrials.gov ID: NCT05402696). Of those lesions which underwent successful EMR, margin STSC was applied systematically aiming to create at least a 2-3mm rim of completed ablated tissue (complete whitening). Recurrence was evaluated both endoscopically, using a standardized protocol for the post-EMR scar, and histologically.

**Results:**

From 06/2022-09/2023, 335 LNPCPs were endoscopically resected, including 209 by EMR. Following successful EMR, 182 (87.1%) underwent margin STSC. Of these lesions, 49 LNPCPs in 46 patients were assessed at first surveillance colonoscopy. Margin STSC was complete for 44 (89.8%) lesions and incomplete for 5 (10.2%) due to difficult angulation/positioning (n=3) and ileocecal valve location (n=2). Median interval to first surveillance colonoscopy was 6 (IQR 6-7) months. There was no evidence of recurrence noted on endoscopy. Biopsy was performed in 44 (89.8%) with no evidence of histologic recurrence.

**Conclusions:**

Thermal ablation of the defect margin with STSC effectively negates recurrence and should be considered standard of care following EMR.

**Funding Agencies:**

None

